# Regulating T Cell Population Alleviates SLE by Inhibiting mTORC1/C2 in MRL/lpr Mice

**DOI:** 10.3389/fphar.2020.579298

**Published:** 2021-01-14

**Authors:** Dongya Zhang, Meiling Wang, Guoping Shi, Peng Pan, Jianjian Ji, Pengfei Li

**Affiliations:** ^1^ Key Laboratory of Inflammation and Immunoregulation, School of Medical and Holistic Integrative Medicine, Nanjing University of Chinese Medicine, Nanjing, China; ^2^ Department of Clinical Laboratory, Jiangsu Province Hospital of Traditional Chinese Medicine, Affiliated Hospital of Nanjing University of Chinese Medicine, Nanjing, China; ^3^ Department of Anesthesiology, Kunshan Hospital of Traditional Chinese Medicine, Affiliated Hospital of Nanjing University of Chinese Medicine, Kunshan, China; ^4^ Jiangsu Key Laboratory of Pediatric Respiratory Disease, Institute of Pediatrics, Affiliated Hospital of Nanjing University of Chinese Medicine, Nanjing, China

**Keywords:** SLE, mTOR, rapamycin, INK128, T cell populations

## Abstract

It’s well known that the mammalian target of rapamycin (mTOR) exerts a critical role in the regulator of immune cells and is associated with T cells dysfunction in patients with systemic lupus erythematosus (SLE). Antigen-induced T-cell proliferation via mTORC1 suppressed by Rapamycin has been used to improve SLE primarily. Previously it has showed that INK128, a highly potent, specific orally inhibitor of mTORC1 and mTORC2, significantly attenuates SLE in pristine-induced lupus mice. Herein we compared the cure effects of INK128 and rapamycin on lupus mice. We treated MRL/lpr mice with INK128 or rapamycin at 12 weeks-age. The effect of the two inhibitors on the lupus mice was determined by immunohistochemistry. The effect of the two inhibitors on T cell populations was investigated by flow cytometry. The mTOR signaling was measured by Western Blot. INK128 remarkably alleviated SLE by reducing splenomegaly, renal inflammation and damage, and resuming T-cell dysfunction. The more effective of INK128 on SLE than rapamycin. INK128 effectively suppressed mTORC1 and mTORC2 activity in T cells, but rapamycin just suppressed mTORC1 activity. Thus, our results show that INK128 is can effectively alleviate SLE and be used as one of the potential clinical therapeutic candidates for SLE.

## Introduction

The systemic lupus erythematosus (SLE) is an autoimmune disease characterized by hyperproliferation and hyperactivation of lymphocytes, autoantibody production (Kim et al., 2017). The involvement of multiple organs and tissues that including the brain, blood, and kidney in patients (Tsokos, 2020). Although the survival rate of patients with SLE has increased significantly over the past decades, current therapy for SLE is still not satisfactory. A clinical need to search for new therapies is necessary for tailoring effective treatment according to patients’ characteristics (Kim et al., 2017).

It was believed that the involvement of immune cell dysfunction and the production of autoantibodies cause SLE (Poissonnier et al., 2016). Previous studies showed that cell dysfunction of T cells, as a key component of the effector and regulatory immune responses, was significantly general in SLE patients. Dysfunction of T helper (Th)1 cells and Th2 cells involve in the development of diffuse proliferative lupus nephritis and crescentic glomerulonephritis in SLE (De la Cruz-Mosso et al., 2018; Mesquita et al., 2018). Emerging evidence that the balance between Th17 and regulatory T cell (Treg) is a new model about the progression of clinical symptoms in SLE patients (Poissonnier et al., 2016; Kubo et al., 2017). Increasing Th17 cells were correlated with flares and organ damages in SLE patients (Schmidt et al., 2015; Kubo et al., 2017). Moreover, Th17 treated with IL-17-blocking antibodies could relieve lupus symptom in SLE (Shah et al., 2010). In contrast, reduction number and defective function of Treg in SLE patients has also been reported in previous studies and target to Treg can also treat SLE (Wang et al., 2017; Kato and Perl, 2018). Taken together, improving T-cell populations dysfunction can be a promising new therapeutic target in SLE patients which has been developed lately in the world (An et al., 2017; Moon et al., 2018).

Several lines of evidence have indicated that many signaling pathways are involved in T-cell populations dysfunction. MTOR signaling pathways played an important role in differentiation, function and activation of T cells (Perl, 2016). MTOR is a ubiquitous serine/threonine kinase, including two interacting complexes mTORC1 and mTORC2, regulates cell growth, proliferation and survival (Lee et al., 2017; Yang et al., 2017). Activation of the mechanistic target of mTOR has emerged as a key driver of abnormal lineage specification within the immune system (Lai et al., 2018), which played an important role in SLE. Inhibition of mTOR activation using rapamycin in the T cells of patients with SLE was identified. Previous study showed rapamycin, which was an inhibitor of mTORC1, improves the clinical outcome in mouse lupus models and in patients with SLE (Lai et al., 2012; Kato and Perl, 2018) via reducing development of Th17 and enhancing development and function of Treg (Kato and Perl, 2018). Most studies indicated mTORC1 drived expansion of Th1/Th17 and inhibited the development of Treg cells in SLE (Perl, 2016; Elahi et al., 2018) (Perl, 2016; Kato and Perl, 2018), while mTORC2 also participated in development and function T cells. Previous studies showed mTORC2 activation could inhibit the development of Treg cells in SLE (Perl, 2016; Kato and Perl, 2018). Activation of mTORC2 exhibited elevated-activation in Treg cells in SLE patients of (Lai et al., 2012; Essig et al., 2017; Kato and Perl, 2018), and inhibition of mTORC2 might promote the development of Treg cells (Perl, 2016; Kato and Perl, 2018). Thus, both inhibition of mTORC1 and mTORC2 may be more efficient in regulation T cell dysfunction in SLE (Perl, 2016; Kato and Perl, 2018).

Our previous studies showed that INK128, which is a selective, highly potent, and orally inhibitor of mTORC1 and mTORC2, attenuates SLE in pristine-induced lupus mice and MRL/lpr mice (Li et al., 2018; Shi et al., 2019a). INK128 (formerly TAK228, MLN0128, also known as sapanisertib) is a selective, highly potent, and orally inhibitor of mTORC1 and mTORC2 which is in phase I and II clinical trials as a single agent and in combination therapy for patients with advanced solid tumors (Koo et al., 2014; Ghobrial et al., 2016). Preclinical studies have shown that INK128 has antitumor activity in multiple tumor types, including prostate cancer, B-cell leukemia, breast cancer, renal cell carcinoma, and bone and soft tissue sarcomas (Hayman et al., 2014; Wilson-Edell et al., 2014; Momcilovic et al., 2015). Previous study also showed INK128 exhibited anti-inflammatory activity in lipopolysaccharide-activated RAW 264.7 cells (Pan et al., 2014). Our recent study also showed INK128 attenuates dextran sodium sulfate-induced colitis (Shi et al., 2019b). In this study we evaluated the therapeutic effects of INK128 on SLE compare to rapamycin in MRL/lpr mice.

In the present study, INK128 was used to improve lupus-symptom in MRL/lpr mice. MRL/lpr mice, which develop a systemic autoimmune disease resembling human SLE, were used (Hao et al., 2018; Kishimoto et al., 2018). INK128 remarkably alleviated SLE by reducing splenomegaly, renal inflammation and damage, and resuming T-cell dysfunction. INK128 has more effective on improving SLE than rapamycin. INK128 effectively suppressed mTORC1 and mTORC2 activity in T cell isolated MRL/lpr mice via reducing phosphorylation of S6K1, 4EBP1 and AKT. However, rapamycin just suppressed mTORC1 activity in T cell isolated MRL/lpr mice via reducing phosphorylation of S6K1 and 4EBP1. Furthermore, *in vitro* experiments confirmed that INK128 had more effectively effects on T cell dysfunction than rapamycin. Taken together, our results indicate INK128 can both inhibit both mTORC1 and mTORC2, which are more effective than rapamycin at preventing of SLE.

## Materials and Methods

### Reagents

INK128 and rapamycin were purchased from Sigma-Aldrich (St Louis, United States). The antibodies used in flow cytometry include antibodies against mouse B220, AA4.1, CD4, CD4, CD25, FOXP3, IFNγ IL-17A and CD69, were purchased from eBiosciences (San Diego, United States). IgG and IgM were analyzed by using mouse IgG and IgM ELISA kits, and Mouse Albumin ELISA Quantitation Set was obtained from Bethyl Laboratories (Montgomery, United States). ELISA kit detecting IL-17A and IFNγ were purchased from Abcam (Cambridge, United States). The immunohistochemistry antibodies C3c was purchased from Abcam (Cambridge, United States). Dulbecco’s modified Eagle’s medium, RPMI 1640, and fetal bovine serum (FBS) were purchased from Thermo Fisher (United States).

### Animal Experiment

Eight-week-old female MRL/lpr mice and C57BL/6 wild-type mice were obtained from the Model Animal Research Center of Nanjing University (Nanjing, China) and kept under pathogen-free and housing conditions in a 12/12 h light and dark cycle. All of the animal experiments were performed in accordance with the National Institutes of Health Guide for the Care and Use of Laboratory Animals, and with the approval of Animal Care and Use Committee of Nanjing University of Chinese medicine. At the beginning of 12 weeks, proteinuria levels were determined once a week. The mice were randomly divided to Control group treated with normal saline treatment (containing 0.5% dimethyl sulfoxide (DMSO), INK128 treatment group and rapamycin treatment group. At 12 weeks of age, MRL/lpr mice started to injection by i.p., two times per week. MRL/lpr mice treated with 1 mg/kg INK128 per kilogram of body weight (Shi et al., 2019a) and rapamycin group treated with 1 mg/kg rapamycin per kilogram of body weight (Shi et al., 2019a). Both of INK128 and rapamycin dissolved in DMSO as a stock solution and then diluted with saline to the indicated concentrations. All mice were sacrificed by cervical dislocation at 27 weeks of age.

### Proteinuria Analysis

Collecting the urine of all mice once a week. Total urinary protein was detected by Mouse Albumin ELISA Quantitation Set according to the manufacturer’s instructions and the urine was applied at dilutions of 1:100. Severe proteinuria was defined as ≥300 mg/dl urinary protein concentration in two consecutive examinations (Ji et al., 2016).

### Histological and Immunohistochemical Analysis

Tissues samples fixed in formalin (Beyotime, Shanghai, China) were cut into 4 μm sections from paraffin-embedded kidney tissue, and stained with hematoxylin and eosin (HE), or periodic acid–Schiff (PAS) (Beyotime). Immunohistochemistry was performed on paraffin-fixed kidney sections by antibodies against C3c. Immunolabeled sections were scanned using an Aperio ScanScope Slide Scanner (BIO-TEK Instruments, Winooski, United States) (Ji et al., 2016).

### Enzyme-Linked Immunosorbent Assay

Serum IgG and anti-IgM were determined with mouse IgG, IgG2a, and IgM ELISA kits according to the manufacturer’s instructions, and the sera used to detection was diluted from 1:100,000 to 1:500,000. The concentration of IL-17A and IFNγ in the serum was measured by ELISA kit according to the manufacture’s recommendations. Serum was used at a 1:4 dilution. Absorbance was determined using an ELx-800 Universal Microplate Reader (BIO-TEK Instruments).

### Flow Cytometry

Briefly, mouse spleen and draining lymph node (dLN) and kidney grinded to cell suspension resuspended in a red blood cell lysing buffer at 37°C for 2 min, followed by passing through a 70 mm cell strainer and centrifuged.

For intracellular IL-17A staining, cells were treated with 5 ng/ml PMA (Invitrogen) and 1 ng/ml ionomycin (Enzo Life Sciences, Inc.) for 5 h and then added at 10 ng/ml brefeldin A (Enzo Life Sciences, Inc.) 30 min before staining with CD4- FITC (eBiosciences, San Diego, United States). Next cells were stained with IL-17A-PE (eBiosciences) followed by permeabilization of the cells with Cytofix/Cytoperm (BD Biosciences, San Diego, United States).

For intracellular Foxp3-PE staining (Pan et al., 2014), cells were permeabilized with Cytofix/Cytoperm, and stained with Foxp3-PE (eBiosciences), followed by stain with CD4-FITC and CD25-APC (eBiosciences). Cells were detected by FACS Calibur Flow Cytometer (Becton Dickinson, San Diego, United States) and analyzed with the Flow Jo software.

### Preparation of CD4^+^ T Cells

CD4^+^ T cells were isolated from the spleen cells by T cell isolation kit (Miltenyi Biotec), and resuspended in RPMI 1640 medium (Gibco Invitrogen) supplemented with 10% FBS (Gibco Invitrogen) (4 × 10^4^ cells/100 ml). Cells were treated with 50 nM INK128 and rapamycin for 2 h before stimulated with 1 mM PMA (Shi et al., 2019a).

### Western Blot Analysis

Western blot analysis was performed as described previously. Cells were lyzed in buffer containing 50 mmol/L Tris–HCl, pH 8.0, 150 mmol/L NaCl, 0.02% NaN_3_, 0.1% SDS, 100 mg/L phenylmethylsulfonylfluoride, 1 mg/L aprotinin, and 1% Triton. Cell extract was separated by SDS-PAGE and transferred onto PVDF membranes (Millipore). Antibodies against P-4EBP1 (1:1,000), P-S6K1 (1:1,000) and P-AKT (1:1,000) for Western blotting were purchased from Cell Signaling Technology. Protein bands were visualized using ECL Plus Western blotting detection reagents (Millipore). GAPDH was used as an internal control.

### Statistical Analysis

Data were statistically evaluated by the non-parametric Mann-Whitney U-test. Reported values are median ± interquartile range as indicated. Comparison between each group was performed using the non-parametric Mann-Whitney U-test. Statistical significance was defined as a confidence interval equal to or greater than 95% or *p* ≤ 0.05. All data was analyzed by using statistical software GraphPad Prism (GraphPad, San Diego, United States).

## Results

INK128 attenuates symptoms of lupus-like disease in MRL/lpr mice.

**FIGURE 1:**
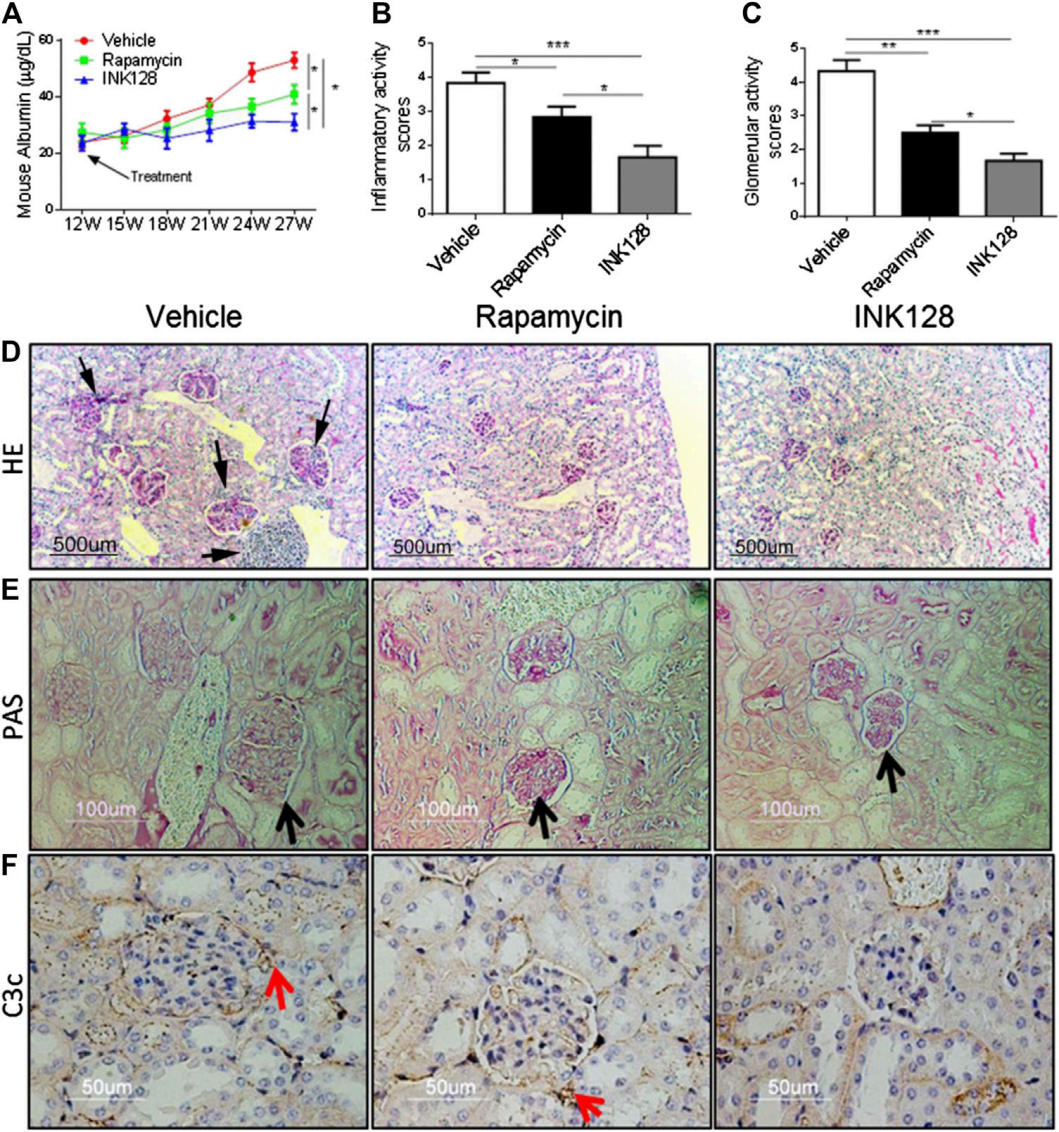
INK128 attenuates symptoms of lupus-like disease in MRL/lpr mice. **(A)** Proteinuria was determined in each experiment using ELISA. Data are the median ± interquartile range from 5 or 6 independent experiments with one mice per group for rapamycin group or other group. Analyzed by the non-parametric Mann-Whitney U-test. **(B–E)** Kidney sections from MRL/lpr mice showed histologic differences. Hematoxylin and eosin **(H,E)** staining revealed an uneven renal cortical surface with inflammatory infiltrates in the vehicle-treated MRL/lpr mice. Periodic acid–Schiff (PAS) staining confirmed the expansion of glomeruli in vehicle-treated MRL/lpr mice, with enlarged glomeruli, distension of tubular lumina, protein casts, and either epithelial or endothelial deposits. **(F)** C3c deposit was analyzed by IHC staining. Data are the median ± interquartile range from 5 or 6 independent experiments with one mice per group for rapamycin group or other group. Analyzed by the non-parametric Mann-Whitney U-test. (*p ≤ 0.05, **p ≤ 0.01, ***p ≤ 0.001). INK128 reduces concentrations of auto-antibodies and inhibits activation of B cells.

MRL/lpr mice possess a rapid and severe form of lupus like disease (Moon et al., 2018). Mice were treated with INK128 and rapamycin from 12 weeks of age. From 15 to 27 weeks of age, vehicle treated mice developed more and more severe proteinuria, while INK128 and rapamycin prevented the onset of proteinuria from 15 weeks of age, and INK128 significantly inhibited the production of proteinuria from the 18th week. INK128 are more effective than rapamycin at preventing to develop proteinuria in the experiment (Figure 1A).

Lupus nephritis or glomerulo-nephritis is the most common manifestation of organ pathology in SLE (Hao et al., 2018). To asses; s whether INK128 can improve renal damage and inflammation, histopathological analysis was conducted. MRL/lpr mice were sacrificed at 27 weeks, H&E or PAS staining of kidney sections showed enlarged glomeruli, with diffuse cellular infiltration and marked interstitial mononuclear cell infiltration, while INK128 and rapamycin both significantly reversed the glomerulonephritis and interstitial inflammatory infiltration (Figures 1D,E). Histologic scoring of the pathologic changes in the kidneys showed INK128 had more significant improvement compared to rapamycin (Figures 1B,C).

Complement is implicated in the pathogenesis of SLE, and C3c, a marker of intra glomerular complement activation in MRL/lpr mice, is deposited in the kidney during lupus nephritis (Karasawa et al., 2018). Our results showed that INK128 significantly reduced the deposition of complement factor C3c, but rapamycin showed slight effects in kidney (Figure 1F). Collectively, these results, together, demonstrated INK128, significantly attenuated disease activity in MRL/lpr mice.

**FIGURE 2:**
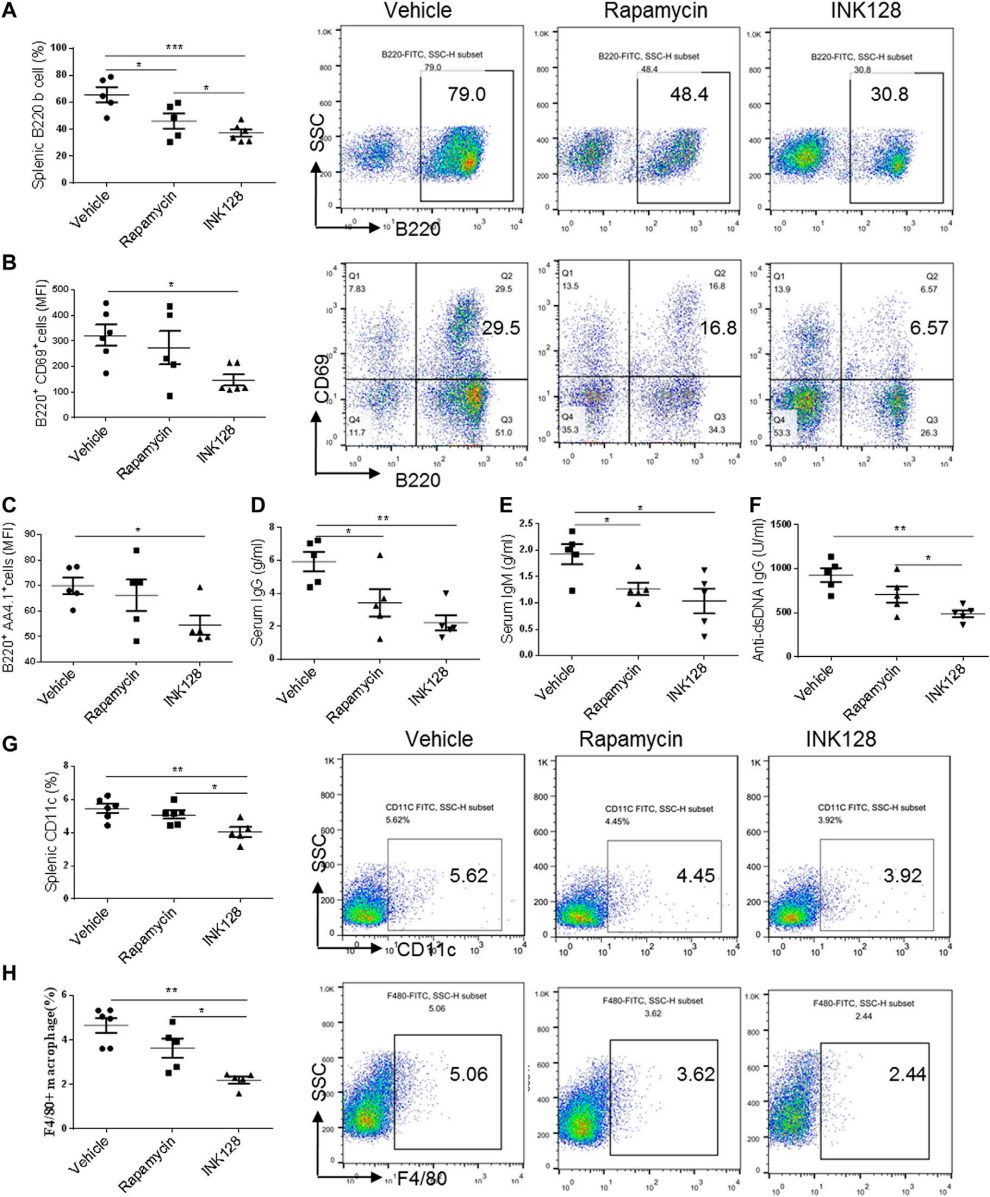
INK128 reduces concentrations of auto-antibodies and inhibits activation of B cells. **(A)** The percentage of B220^+^ B cells in spleens were analyzed by flow cytometry. Data are representative or are the median ± interquartile range from five or six independent experiments with one independent mice per group in each experiment. **(B)** The surface expression of CD69 in B220^+^ B cells in the spleens were analyzed by flow cytometry. Data are representative or are the median ± interquartile range from five or six independent experiments with one independent mice per group in each experiment. **(C)** The surface expression of B220^+^ AA4.1^+^ B cells in the spleens were analyzed by flow cytometry. Data are representative or are the median ± interquartile range from five or six independent experiments with one independent mice per group in each experiment. **(D–F)** Serum levels of total IgG, IgM and anti-dsDNA IgG were determined by ELISA. Data are representative or are the median ± interquartile range from five independent experiments with one or two independent mice per group in each experiment. **(G,H)** The percentage of CD11c^+^ DC cells **(G)** and F4/80^+^ macrophage cells **(H)** in spleens was analyzed by flow cytometry. Data are representative or are the median ± interquartile range from five independent experiments with one or two independent mice per group in each experiment. Data was analyzed by the non-parametric Mann-Whitney U-test. (*p ≤ 0.05, **p ≤ 0.01, ***p ≤ 0.001). Immunomodulatory effects of INK128 treatment on T cell populations in MRL/lpr mice.

To determine whether INK128 effectively inhibits B cells, splenic B cell subpopulations were evaluated by flow cytometric analysis. In MRL/lpr mice, INK128 significantly decreased the percentage of total B cells (Figure 2A) and reduced the activation of B cells, represented by suppressing expression of CD69 (Figure 2B). INK128 also reduced the percentage of mature B cells, which is characterized by AA4.1 expression (Figure 2C). Moreover, percentages of B cells were also significantly reduced after rapamycin treatment, but INK128 showed more effective than rapamycin at suppressing B cells. B cells are the main sources of auto-antibody, and then the levels of auto-antibodies were measure after INK128 and rapamycin treatment. The results showed INK128 decreased the amount of IgG, which is known to activate complement in lupus nephritis (Figure 2D). INK128 also reduced plasma IgM and anti-dsDNA IgG levels (Figures 2E,F). Rapamycin also displayed well effects on IgG and anti-dsDNA IgG production, but INK128 showed higher effective on reduction of anti-dsDNA IgG in MRL/lpr mice.

DCs and macrophages contribute to the inflammation progress in MRL/lpr mice. Then we examined percentage of DCs and macrophages in spleen of MRL/lpr mice, and results showed that INK128 suppressed percentages of both DCs and macrophages in spleens (Figures 2G,H). However, rapamycin showed no effects on suppressing percentages of macrophages and DCs.

**FIGURE 3:**
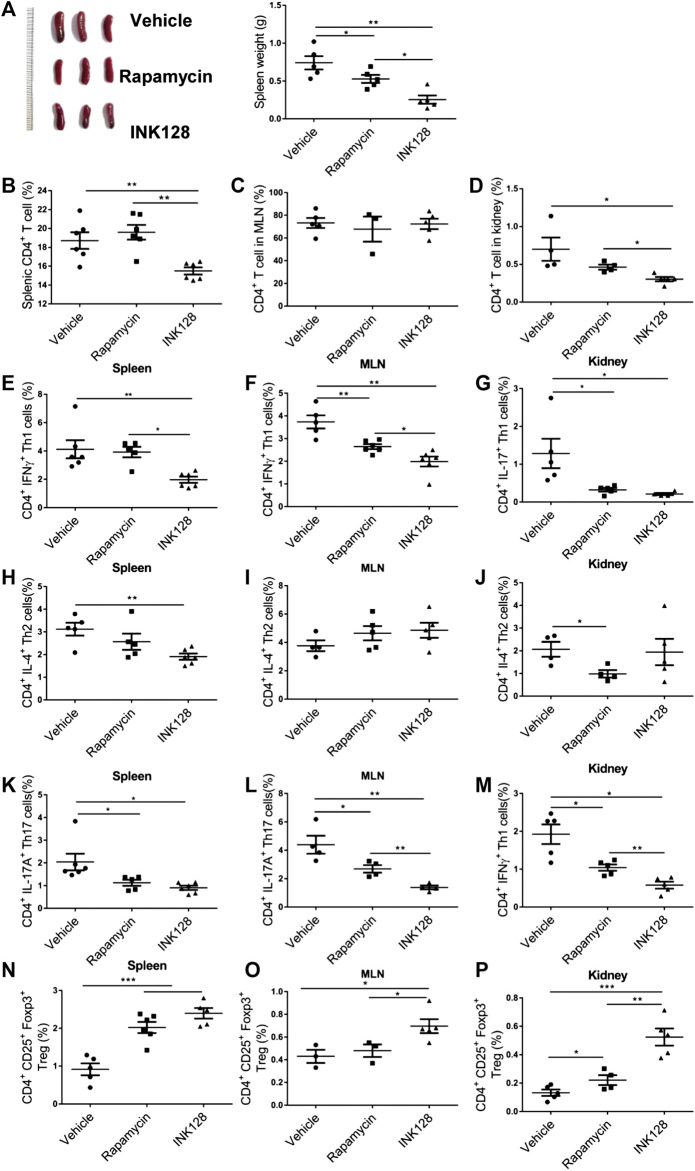
Immunomodulatory effects of INK128 treatment on T cell populations in MRL/lpr mice. **(A)** Representative photographs of spleens from each group (left). Weight of spleens from each groups. Data are representative or are the median ± interquartile range from 5 or 6 independent experiments with one mice per group in each experiment. **(B–D)** The percentage of CD4^+^ T cells in spleens, MLN and kidney were analyzed by flow cytometry (n = 6). **(E–G)** The percentage of CD4^+^ IFNγ^+^ Th1 cells in spleens, MLN and kidney were analyzed by flow cytometry. Data are representative or are the median ± interquartile range from 5 or 6 independent experiments with one mice per group in each experiment. **(H–J)** The percentage of CD4^+^ IL-4^+^ Th2 cells in spleens, MLN and kidney were analyzed by flow cytometry. Data are representative or are the median ± interquartile range from 5 or 6 independent experiments with one mice per group in each experiment. **(K–M)** The percentage of CD4^+^ IL-17A^+^ Th17 cells in spleens, MLN and kidney were analyzed by flow cytometry. Data are representative or are the median ± interquartile range from 5 or 6 independent experiments with one mice per group in each experiment. **(N–P)** The percentage of CD4^+^ CD25^+^ Foxp3^+^ Treg cells in spleens, MLN and kidney were analyzed by flow cytometry. Data are representative or are the median ± interquartile range from 5 or 6 independent experiments with one mice per group in each experiment. Data was analyzed by the non-parametric Mann-Whitney U-test. (*p ≤ 0.05, **p ≤ 0.01, ***p ≤ 0.001.). INK128 suppresses mTORC1 and mTORC2 activity in CD4^+^ T cells in MRL/lpr mice.

Subsequently, immune system of MRL/lpr mice was monitored. We first analyzed splenomegaly in all groups of mice, and results showed the splenomegaly was alleviated in INK128 and rapamycin-treated mice (Figure 3A). Obviously, INK128 displayed better improvement than rapamycin at splenomegaly in MRL/lpr mice (Figure 3A). A massive proliferation of T cells is characteristic for MRL/lpr mice, and we investigated change of T cell populations in spleens, MLN and kidney of MRL/lpr mice after INK128 and rapamycin treatment. CD4 T cells form a large proportion of the inflammatory cells participate in diseased MRL/lpr mice. Our results showed INK128 significantly reduced percentage of CD4 T cells in spleen and kidney (Figures 3B–D).

Upon antigenic stimulation, naïve CD4^+^ T cells activate and differentiate into various helper T cells including Th1, Th2, Th17 and Treg cells (Sugita et al., 2017). Th1 cells had been considered to play an important role in the development of SLE (De la Cruz-Mosso et al., 2018). In our results, INK128 significantly decreased percentage of Th1 in spleen, MLN and kidney, and rapamycin also reduced numbers of Th1 in MLN and kidney (Figures 3D–F). However, INK128 are more effective on reducing Th1 cells in MRL/lpr mice. Our results also showed INK128 significantly decreased percentage of Th2 in spleen, while rapamycin reduced numbers of Th2 in kidney (Figures 3H–J). Th17 cells were increased and played pathogenic roles in SLE (De la Cruz-Mosso et al., 2018). Our results showed that INK128 and rapamycin both decreased percentages of Th17 cells in spleen, MLN and kidney, while INK128 displayed more efficient effects on reducing Th17 cells in MLNs (Figures 3K–M). Circulating Treg cells decrease during disease progress in SLE, and the immune suppressive function of Treg cells was impaired (Ferreira et al., 2017). In our study, INK128 can recover percentage of Treg cells in spleen, MLN and kidney, while rapamycin just increased the percentages of Treg cells in spleens and kidneys (Figures 3N–P). Moreover, INK128 displayed more efficient effects on recovering Treg cells in spleen, MLNs and kidney (Figures 3N–P). In summary, these results further confirmed INK128 could regulate the T cell populations in MRL/lpr mice, and INK128 showed more efficient effects on modulating T cells than rapamycin.

**FIGURE 4:**
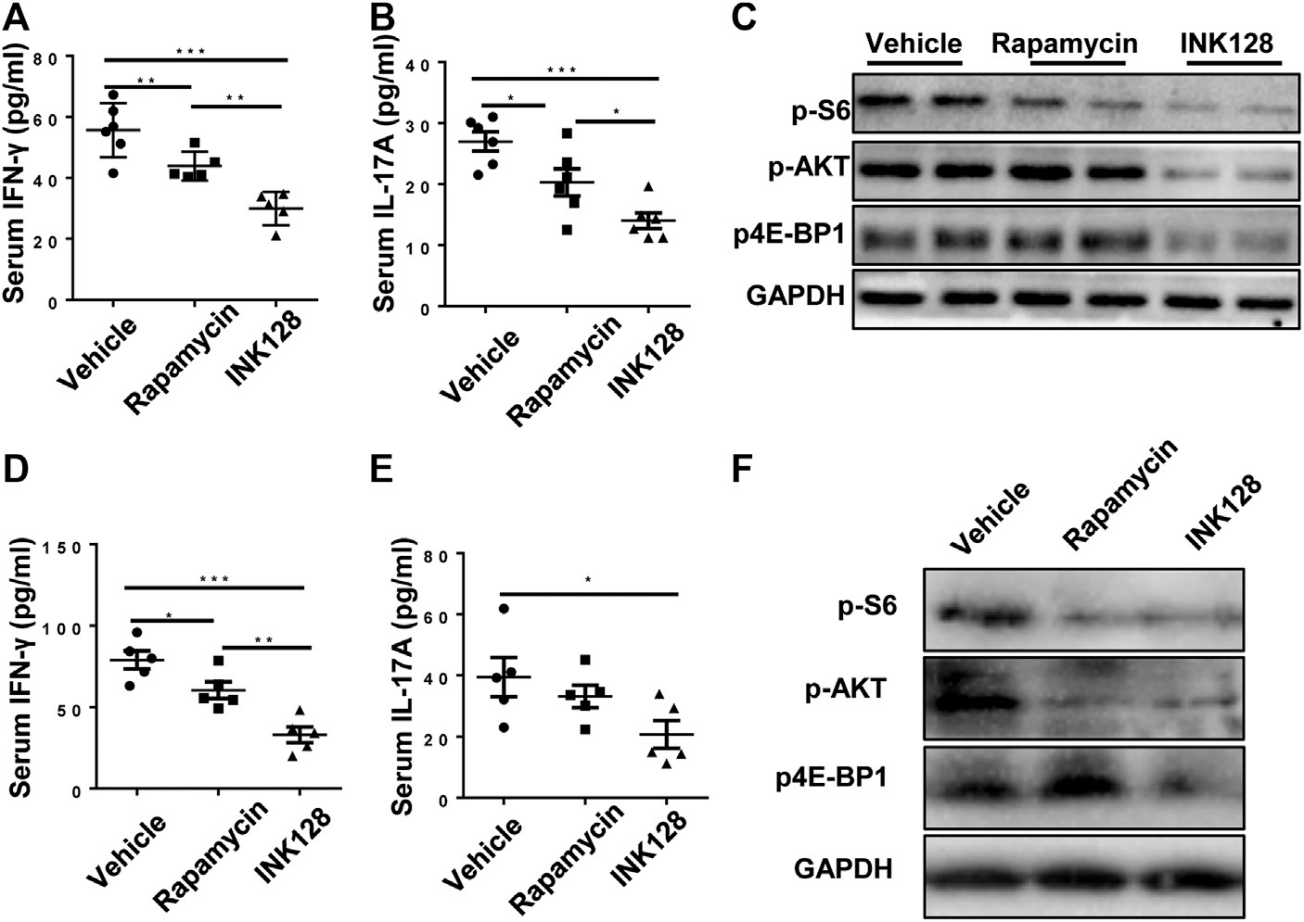
INK128 suppresses mTORC1 and mTORC2 activity in CD4^+^ T cells in MRL/lpr mice. **(A,B)** Serum levels of IFNγ and IL-17A were determined by ELISA. Data are representative or are the median ± interquartile range from 5 or 6 independent experiments with one mice per group in each experiment. **(C)** Immunoblot analysis of phosphorylated of 4EBP1, S6K1 and AKT in lysates of CD4 T cells in MRL/lpr mice. **(D,E)** Culture supernatant of IFNγ and IL-17A were determined by ELISA. Data are representative or are the median ± interquartile range from 5 or 6 independent experiments with one mice per group in each experiment. **(F)** Immunoblot analysis of phosphorylated of 4EBP1, S6K1 and AKT in lysates of CD4 T cells. Data are representative or are the median ± interquartile range from 5 or 6 independent experiments with one mice per group in each experiment. Data was analyzed by the non-parametric Mann-Whitney U-test. (*p ≤ 0.05, **p ≤ 0.01, ***p ≤ 0.001).

Our results showed that INK128 dramatically changed percentages of Th1, Th2, Th17 and Treg cells. Previous studies showed IFNγ and IL-17A, derived from Th1 and Th17 cell respectively, played a pathology role in lupus progress in MRL/lpr mice (Schmidt et al., 2015). The levels of IFNγ and IL-17A in serum in MRL/lpr mice were examined, and results showed INK128 and rapamycin both reduced the concentrations of IFNγ and IL-17A in the serum in MRL/lpr (Figures 4A,B), while INK128 showed more efficient effects on reducing IFNγ and IL-17A. To determine whether the effect of INK128 on regulation of T cells in MRL/lpr mice were attributed to mTOR activation, we examined mTOR activity in CD4 T cells. CD4 T cells were isolated from spleen in MRL/lpr mice after INK128 and rapamycin treatment, and mTOR activity was examined using WB. Results showed INK128 and rapamycin treatment both blocked the phosphorylation of the 4E-BP1 and S6K1, which were substrate of mTORC1, while INK128 could also reduce levels of phosphorylation of AKT, which were substrate of mTORC2 (Figure 4C).

Furthermore, untouched and fresh splenic CD4 T cells were isolated from spleen of MRL/lpr mice, and then the CD4 T cells were cultured for 3 d in the presence of anti-CD3/CD28, INK128 or rapamycin. Subsequently, levels of IL-17A and IFNγ in the cultures were measured by ELISA, and INK128 and rapamycin significantly reduced the concentrations of IFNγ and IL-17A in the culture, while INK128 displayed more efficient effects on reducing IFNγ and IL-17A (Figures 4D,E). Moreover, INK128 and rapamycin both inhibited mTORC1 in T cells based on pS6K1 and p-4EBP1 expression detected by immunoblotting (Figure 4F). INK128 also inhibited mTORC2 in T cells based on p-AKT expression (Figure 4F).

## Discussion

SLE is an autoimmune disease with persistent inflammation which damages multiple organs (An et al., 2017). SLE is initiated by destroying immune tolerance to self. Recent reports showed that elevation of T cell populations were thought to be major factors correlated with lupus-symptom in SLE patients (Shah et al., 2010; Poissonnier et al., 2016; An et al., 2017). Activation of the mTOR pathway underlies the pathogenesis of SLE and mTOR contribute to immune disorder in SLE (Perl, 2016; Kato and Perl, 2018). mTOR played an important role in T cells dysfunction (Perl, 2016; An et al., 2017).

The mechanistic target of rapamycin (mTOR) is a ubiquitous serine/threonine kinase that plays pivotal roles in integrating growth signals. To support proliferation and survival under stress, two interacting complexes that harbor mTOR, mTORC1 and mTORC2, promote the transcription of genes involved in carbohydrate metabolism and lipogenesis, enhance protein translation (Lee et al., 2017; Tsang et al., 2018). MTORC1 is activated by nutrients and the availability of cellular energy, such as amino acids and ATP11. In turn, growth factors (e.g., insulin) stimulate mTORC1 via the tuberous sclerosis complex (TSC). Upon activation, mTORC1 controls protein synthesis by inducing mRNA translation (Lee et al., 2017). In addition to responding to growth signals and promoting cell proliferation, mTORC1 is also actively involved in blocking autophagy, a complex lysosomal degradation pathway that allows cell survival during starvation (Wilson-Edell et al., 2014; Lee et al., 2017). Activation of mTORC1 precedes the onset of SLE (Perl, 2016). Targeting mTORC1 over-activation with rapamycin provides an opportunity to supplant current therapies with severe side effect profiles. The activation of mTORC1 preceded disease flares by 4 months. MTORC1 also drives the pro-inflammatory expansion of Th1 and Th17. MTORC1 inhibits the development of TREG cells via reducing expression of the Foxp3 (Perl, 2016). Then target to mTORC1 supplemented a method to improve lupus, and previous studies showed rapamycin blocked the pro-inflammatory, Th17 cells and expands Tregs via mTORC1 in SLE (Perl, 2016; Kato and Perl, 2018). Another mTORC1 inhibitor NAC also had well effects on lupus symptom in SLE (Lai et al., 2012).

Although mTORC2 regulation is less well understood, Treg cells exhibited increased activities of mTORC2 in SLE, and mTORC2 inhibit the development of Treg cells (Kato and Perl, 2018). Recent study found prolonged rapamycin treatment enabled blockade of mTORC2, and increased percentage of Treg in SLE (Kato and Perl, 2018). Curiously, previous study also found rapamycin treatment reduced mTORC1 and enhanced mTORC2 activities in T cells *in vitro* (Kato and Perl, 2014). Therefore, what effects of rapamycin on mTORC2 remained unclear in SLE. Consequently, it is necessary to find a mTOR kinase inhibitor, which fully block mTORC1 and mTORC2 in SLE.

INK128 is a selective, highly potent, and orally inhibitor of mTORC1 and mTORC2 which is in phase I and II clinical trials as a single agent and in combination therapy for patients with advanced solid tumors (Hayman et al., 2014; Ghobrial et al., 2016). Our recent results showed INK 128 attenuates SLE in pristine-induced lupus mice and MRL/lpr mice by regulating inflammation-induced CD11b^+^Gr1^+^ cells (Li et al., 2018; Shi et al., 2019a). Previous study also showed INK128 exhibited anti-inflammatory activity in lipopolysaccharide-activated RAW 264.7 cells. INK128 strikingly inhibited the phosphorylation of p70S6K, 4E-BP1 and AKTSer473 in both unstimulated and LPS-stimulated cells (Pan et al., 2014), and this study suggested INK might display anti-inflammatory effect. In our result, INK128 remarkably alleviated SLE by reducing splenomegaly, renal inflammation and damage, and resuming T-cell dysfunction. INK128 has more effective on improving SLE than rapamycin. INK128 effectively suppressed mTORC1 and mTORC2 activity in T cell isolated MRL/lpr mice via reducing phosphorylation of S6K1, 4EBP1 and AKT. However, rapamycin just suppressed mTORC1 activity in T cell isolated MRL/lpr mice via reducing phosphorylation of S6K1 and 4EBP1. Furthermore, *in vitro* experiments confirmed that INK128 had more effectively effects on T cell dysfunction than rapamycin. Taken together, our results indicate INK128 can both inhibit both mTORC1 and mTORC2, which are more effective than rapamycin at preventing of SLE.

Based on previous studies and our results, we found that INK128 showed well improvement on lupus symptom in MRL/lpr mice. INK128 regulated T cells populations via blocking both mTORC1 and mTORC2 activity in T cells of MRL/lpr mice. In summary, our findings indicate that INK128 may a good choice for therapeutic approaches of lupus and hope to alleviation of the disease in lupus patients.

INK128 has an important role in resuming T-cell dysfunction and attenuating the development of SLE in MRL/lpr mice, and hence impeding disease progression. The results suggest INK128 may be a potential therapeutic candidate for the treatment of SLE.

## Data Availability Statement

All datasets presented in this study are included in the article/Supplementary Material.

## Ethics Statement

The animal study was reviewed and approved by Animal Care and Use Committee of Nanjing University of Chinese medicine.

## Author Contributions

DZ and PP conceived, designed the experiments, and drafted the article; DZ and PP performed experiments, data analysis and wrote the manuscript; MW performed experiments; GS performed the analysis of flow cytometer. PL supported the materials; JJ provided the concept and design of this study. PL and JJ co-designed experiments and co-wrote the manuscript. All authors reviewed and approved the manuscript final version.

## Conflict of Interest

The authors declare that the research was conducted in the absence of any commercial or financial relationships that could be construed as a potential conflict of interest.

## Funding

This work was supported by the Natural Science Foundation of Jiangsu Province (BK20180825) and National Natural Science Foundation of China (31872732) and the Developing Program for High-level Academic Talent in Jiangsu Hospital of TCM (NO.y2018rc38).

## Abbreviations

SLE, systemic lupus erythematosus; Th, T helper; TREG, regulator T cell; IFN, interferon; IL-17A, interleukin-17A; MLN, mesenteric lymph nodes. S6K1, ribosomal protein S6 kinase beta-1; 4EBP1, eukaryotic translation initiation factor 4E-binding protein 1; AKT, a serine/threonine-specific protein kinase; DCs, dendritic cells.
